# Protein Fractions as Indicators of Stress in Alpacas

**DOI:** 10.3390/ani15131864

**Published:** 2025-06-24

**Authors:** Monika Budzyńska, Joanna Kapustka, Anna Stępniowska

**Affiliations:** 1 Department of Animal Ethology and Wildlife Management, University of Life Sciences in Lublin, 20 950 Lublin, Poland; monika.budzynska@up.lublin.pl; 2 Department of Biochemistry and Toxicology, University of Life Sciences in Lublin, 20 950 Lublin, Poland; anna.stepniowska@up.lublin.pl

**Keywords:** alpaca, shearing, stress response, protein fractions

## Abstract

Shearing can cause a stress response in alpacas with consequences including behavioral, neuroendocrine, and metabolic changes to maintain homeostasis. The use of alternative stress indicators, other than the most common, such as behavior, cortisol, or heart rate, can give a broader insight into the alpaca’s adaptive mechanisms and welfare level. Protein fractions are one of these indicators. A few studies have examined protein fractions as stress indicators in other animal species, showing both acute and long-term changes in the levels of these indicators. The aim of the study was to determine the effectiveness of using blood protein fractions and total protein as indicators of alpacas’ stress response to shearing procedures. It was hypothesized that the total protein and protein fraction levels would change after shearing. In this study in Poland, 20 Huacaya alpacas were examined. Blood samples were taken four times: three days before shearing, on the shearing day, and the fifth and tenth days after shearing. The total protein levels, albumin, and globulins were compared on individual days of the study. The level of total proteins and α1- and ß1-globulins decreased significantly on the fifth day after shearing, compared to the levels three days before shearing. Shearing alpacas induced adaptive responses. The physiological reaction of sheared alpacas can be connected to their fleece removal and the need for thermoregulatory adaptation.

## Simple Summary

As an adaptive mechanism, stress response to shearing can evoke a series of changes in animals’ biological functioning, including alterations in behavioral and hormonal reactions as well as protein metabolism. The level of knowledge about alpaca stress indicators, other than behavior, cortisol, or heart rate, is still limited. The use of alternative stress indicators can provide a broader insight into an alpaca’s adaptation and welfare level. The aim of the study was to determine the effectiveness of blood protein fractions and total protein as indicators of alpacas’ stress response to shearing procedures. In Poland, twenty adult Huacaya alpacas were examined. There were four sampling days: three days before shearing, the shearing day, and the fifth and tenth days after shearing. Total protein, albumin, and globulin levels were determined. The total protein and protein fractions can be used to determine the stress response in alpacas, particularly α1- and ß1-globulins, whose levels were significantly lower on the fifth day after shearing compared to the level before shearing. Shearing induced adaptive responses in alpacas. The physiological reaction of sheared alpacas can be connected to their fleece removal and the need for thermoregulatory adaptation.

## 1. Introduction

The alpaca (*Vicugna pacos*) is an herbivorous ruminate mammal from the camelid family (*Camelidae*). With the development of alpaca breeding and farming in Poland and across Europe, there is a growing need to expand knowledge about this species, which is still low compared to other farm animals. In particular, issues related to assessing their welfare when they are kept outside their domestication region require more detailed knowledge. Alpacas are exposed to stressors associated with veterinary and husbandry procedures [1,2,3]. The shearing procedure can frequently cause a stress response in animals with the development of behavioral, neuroendocrine, and metabolic changes to maintain homeostasis [2,3,4,5,6]. Shearing protects alpacas from heat stress in summer, but the animals must develop responses immediately after the procedure to maintain homeostasis [4,5,7]. A widely used physiological stress indicator in animals is cortisol. Measuring cortisol levels has been used as an effective and objective stress assessment method in alpacas [8,9]. Different biological materials have been used to determine cortisol levels or its metabolites, e.g., blood [10], saliva [8,9], and feces [11]. However, some authors, e.g., MacDougall-Shackleton et al. [12], stated that it is not completely accurate to consider glucocorticoids as a good indicator of stress. Glucocorticoids are only one component of a complex set of physiological and behavioral reactions to stressors; hence, there is a need to develop alternative stress assessment tools. The level of knowledge about alpaca stress indicators, different from behavior, cortisol, or heart rate, is still limited. However, the latest study of Budzyńska et al. [3] indicated the role of other neuroendocrine indicators (noradrenaline and thyroxine) as mid-stress symptoms in the physiological response to shearing in alpacas, making this study innovative and suggesting novel parameters for the stress assessment of these animals. In this earlier study [3], a decreased level of thyroxine was found a few days after shearing, and this finding could be associated with the current research, as thyroxine is a metabolic hormone involved in animal thermoregulation [13]. The use of alternative stress indicators to behavior, cortisol, and heart rate can provide a broader insight into an alpaca’s adaptive mechanisms and welfare level.

Although shearing is well-known as a husbandry practice that induces an acute stress response, it can also affect the thermal homeostasis of the sheared animal [3,13]. This management practice includes removing the fleece, which protects the animal against environmental factors [13]. Some studies reported that fleece removal increases the sensitivity of animals to thermal stress [4,5]. In the case of shearing, animals must adapt to a new situation after fleece removal. The physiological adaptation of sheep to shearing has been studied in the field of thermal homeostasis, according to the alteration of the sheared individual’s thermoregulation [4,6,14,15,16]. Fleece removal results in alterations in protein metabolism in relation to an animal’s exposure to thermal stress [17].

A few studies have examined protein fractions as stress indicators in other animal species, showing both acute and long-term changes in their levels. It is known that protein fractions are a complex non-homogeneous group of proteins that have many functions in the body [18]. Changes in blood total protein and its fractional distribution are commonly the first symptoms of homeostasis disturbance [19]. Dairy cattle and sheep show a decline in blood protein fractions immediately after calving [20,21]. In goats, the level of α1-globulins did not change significantly after parturition, while the α2- and ß1-globulins increased [22]. In calves, the level of cortisol, γ-globulins, and body weight after transport were determinants of health and the subsequent risk of pneumonia [23]. The study of Chorfi et al. [24] indicated that protein fractions show little variability during the day, which may make them a more objective indicator of stress levels. Differently, cortisol shows a diurnal variability, which requires consistently sampling at the same time each day.

The aim of the study was to determine the effectiveness of using blood protein fractions and total protein as indicators of alpacas’ stress response to shearing. The shearing treatment involves different stressors: handling, separation, and restraint, as the procedures directly connected with the shearing event, and the lack of fleece, as the consequence for a sheared animal. It was hypothesized that the total protein and protein fraction levels would change after shearing.

## 2. Materials and Methods

In the study, 20 Huacaya alpacas (10 males and 10 females) aged 3–11 years (younger than 6 years, *n* = 10, and older than 6 years, *n* = 10) were examined. None of the tested females were pregnant or lactating. The animals were kept on a farm in Poland in a semi-intensive system with access to pastures during the day (from 9 am to 4 pm). The rest of the day was spent in the livestock building in group boxing. Males and females were kept separately in the building’s boxes as well as at pasture. The average area per individual in the boxes was 4 m^2^. The pasture was divided into quarters of 3000 m^2^ each. The herd had access to water and hay ad libitum. In addition, a mixed feed concentrate based on alfalfa was provided for the alpacas twice daily, at 150 g/individual/day. The animals had everyday contact with the caretaker and the owners and were accustomed to being touched. They underwent regular health assessments by a veterinarian. The alpacas are sheared once a year, in late spring (May–June), and the shearing procedure is conducted by a professional shearer, who is unfamiliar to the animals.

The study was conducted in May 2023 on four sampling days. Blood samples were taken each sampling day to determine the total protein (TP), albumins (ALB), and α1- (α1-GLB), α2- (α2-GLB), and β1- globulin (β1-GLB) levels. The first sampling day was planned before shearing (to compare responses before and after the procedure); the samples were taken three days before shearing (not just one day before) to decrease the potential anxiety of the animals due to procedures connected with blood collection, e.g., animal capture and restraint while taking the blood sample, and to provide the animals 2 days without any intervention before shearing. The blood collection on days 5 and 10 after shearing was performed to identify whether the shearing procedure influenced the studied indicators over a longer period. On the first sampling day, three days before shearing (3d before), blood samples were taken at 10 am from the right jugular vein by the veterinarian into an anticoagulant test tube (10 mL). On the day of shearing (day of shearing), blood samples were taken 20 min after each individual was sheared. The shearing procedure was performed on all alpacas on the same day starting at 7 am, and it took about 5 h (approximately 12 min for each individual) to complete the shearing on the studied alpacas. The shearing order was random. The average temperature during the day was 22 °C, and it was moderately cloudy, with a mild wind and no rain during the study period. Similar weather conditions occurred on all sampling days. The same blood sampling procedure was conducted on the fifth (5d after) and tenth days (10d after) after the shearing. A detailed description of the shearing and sampling procedures was provided in our earlier study [3]. The sample collection design followed the previous research in which the behavioral and physiological responses in sheep to shearing were examined [15]. After collection, the blood samples were stored at a refrigerated temperature (4 °C) and transported to the laboratory. Samples were centrifugated (3000× *g* × 10 min), pipetted into Eppendorf tubes, and frozen (−80 °C) until analysis.

The plasma samples were tested for total protein, albumins, and α1-, α2-, and β1-globulin levels. Analysis was conducted using immunoenzymatic ELISA kits (Shanghai Qayee Bio-Technology Co., Ltd., Shanghai, China) for total protein (QY-E120122), albumin (QY-E120123), α1-globulin (QY-E120124), α2-globulin (QY-E120125), and β1-globulin (QY-E120126), according to the manufacturer’s instructions.

The results were statistically analyzed using the Statistica program 13.1 (StatSoft, Cracow, Poland). The results were presented in the form of descriptive statistics with the median (Med.) and the lower (Q1) and upper (Q3) quartiles. Extreme and outlier values were excluded from the statistical analysis. The Shapiro–Wilk normality test was performed. The Friedman test was performed to compare the results in the particular sampling days of the study. *p* values < 0.05 were considered significant.

## 3. Results

**Figure 1:**
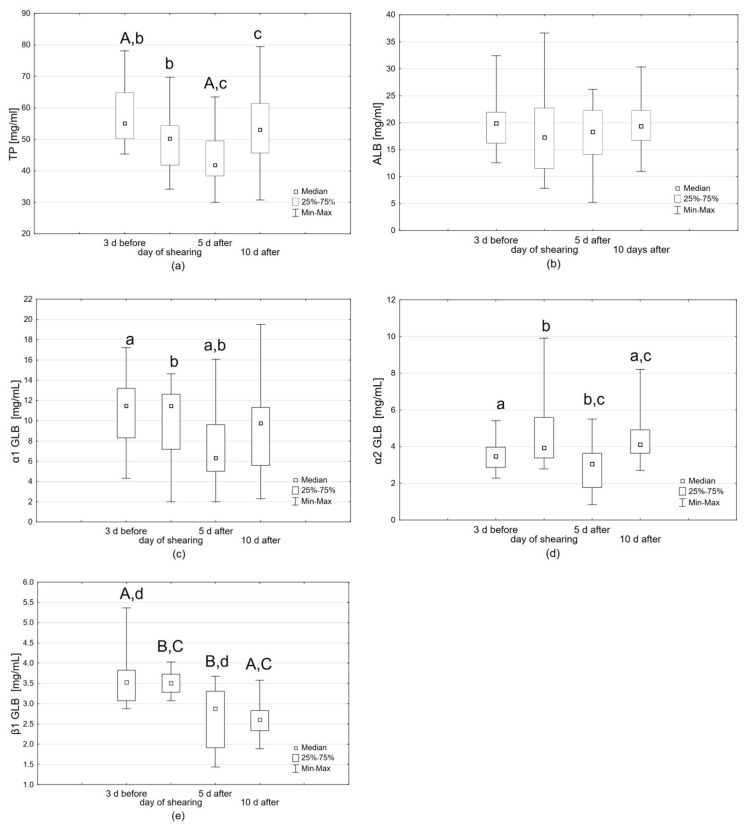
The level of the studied proteins on individual days of the study: (**a**) total protein, A—values marked with the same uppercase letter differ significantly at *p* < 0.001, b,c—values marked with the same lowercase letters differ significantly at *p* < 0.05, (**b**) albumin, (**c**) α1-globulins, a,b—values marked with the same lowercase letters differ significantly at *p* < 0.05, (**d**) α2-globulins, a,c—values marked with the same lowercase letters differ significantly at *p* < 0.05, (**e**) ß1-globulins, A,C—values marked with the same uppercase letters differ significantly at *p* < 0.001, d—values marked with the same lowercase letter differ significantly at *p* < 0.05.

Variability in the individual parameters was observed depending on the day of the study, especially the total protein and α1-, α2-, and ß1-GLB (Figure 1). The level of the total protein on the day of alpaca shearing and on the fifth day after shearing decreased significantly compared to the level three days before the shearing (respectively: t = 2.533, *p* = 0.016; t = 4.523, *p* < 0.001); then, it increased significantly on the tenth day after shearing compared to the fifth day after shearing (t = −2.589, *p* = 0.014) (Figure 1a). The albumin levels did not differ significantly between the individual study days (Figure 1b) (*p* = 0.576). The level of α1-globulin was also significantly decreased on the fifth day after shearing compared to the level three days before shearing (t = 3.208, *p* = 0.003) and between the level on the day of shearing and on the fifth day after shearing (t = 2.221, *p* = 0.033) (Figure 1c). The level of α2-globulin showed significant differences between the tests three days before shearing and ten days after shearing (Z = 2.527, *p* = 0.012), between the level on the day of shearing and five days after shearing (Z = 2.912, *p* = 0.004), and between the fifth and tenth day after shearing (Z = 2.959, *p* = 0.003) (Figure 1d). The level of ß1-globulin was significantly higher three days before shearing and on the day of shearing compared to the following days. The ß1-globulin level was significantly lower on the fifth (Z = 3.049, *p* = 0.002) and the tenth day after shearing (Z = 3.550, *p* < 0.001) compared to the test three days before shearing, as well as on the fifth (t = 4.425, *p* < 0.001) and the tenth day after shearing (t = 7.318, *p* < 0.001) compared to the shearing day (Figure 1e).

## 4. Discussion

The shearing procedure’s impact on the animals involves different types of stressors that can induce several stress responses according to their mechanism and timing. Shearing could be a stressful experience for alpacas due to the handling, separation, and restraint during the shearing process. These procedures are a potential source of acute stress for animals [6]. Moreover, shearing can also induce a stress response because of the fleece removal. Our study indicated that the total protein level and some protein fractions showed variability on individual days of the study. Marai et al. [17] and Piccione et al. [4] suggest that the removal of the fleece evokes changes in the protein metabolism due to the animal’s exposure to thermal stress. Moreover, the alterations in the blood protein level could be caused by a fluid shift between different compartments, playing an essential role in physical protection from the temperature [4]. We found that the total protein level, α1-globulins, and ß1-globulins decreased on the days following shearing. The results from ewes showed a significant effect of shearing and time on the total protein, and a decrease in this indicator was observed over a longer period [4]. The impact of shearing on protein fractions was also studied in sheep; however, the timing of blood collection was different from our study: before shearing and 5 and 60 min after shearing. An increase in α-globulins and ß1- and ß2-globulins was observed within 60 min [6]. The authors of the research on sheep noted that these findings could suggest an acute phase protein reaction as an integral contribution of the acute stress response of ewes [6]. In the present study, the levels of α1-, α2-, and ß1-globulins did not differ significantly before and 20 min after shearing, and an apparent decrease in the levels was observed during the next few days after shearing. The decrease in total protein and protein fraction levels is probably connected with the body’s physiological response to removing the fleece and the need for thermal adaptation. This was also observed in earlier studies on sheep [4,15,25].

The total protein levels (41.85–55.11 mg/mL in this study) were similar to those for adult alpacas: 52.5–75.7 g/L [26], 5.7–7.2 g/dL [27], and 60.7 ± 5.9 g/L (in females) [28]. The albumin levels (17.26–19.87 mg/mL in this study) were slightly lower, as the reference intervals are 31.1–44.2 g/L [26], 2.9–4.3 g/dL [27], and 37.8 ± 3.9 g/L (in females) [28]. The reference values for globulins are given together without division into fractions; therefore, comparing them is impossible. In a study on 30 adult camels [29], the level of α2-globulins was similar (3.05–4.11 mg/mL in this study and 2.4–4.1 g/L in camels), the level of α1-globulin was higher in the present study (6.31–11.47 g/mL in this study and 1.8–2.8 g/L in camels), while the ß1-globulin was lower (2.61–3.53 mg/mL in this study and 8.5–11.2 g/L in camels).

Based on the differences observed in the samples taken 5 and 10 days after shearing, we can state that temporal changes in some protein fractions, especially α1- and ß1-globulins, suggest the animals’ adaptive responses to the thermal stress caused by the lack of fleece. Our study showed some significant changes in the alpacas’ protein indicators; however, it has some limitations. The experiment was limited by the restricted number of animals studied and the number of sampling days. Future studies are needed to perform a detailed analysis of the potential changes in blood total protein concentration and its fractions according to a more frequent blood sampling schedule shortly after shearing. It would be interesting to observe whether the particular indicators could also be considered to be related to acute stress symptoms in alpacas as well as long-term signs of their thermoregulatory abilities.

## 5. Conclusions

Total protein and protein fractions may be used to determine the stress response in alpacas, particularly α1- and ß1-globulins, whose levels were significantly lower on the fifth day after shearing compared to the level before shearing. These protein fractions are promising as stress indicators in alpacas. The physiological reaction of sheared alpacas can be connected to their fleece removal and the need for thermoregulatory adaptation. There are limited studies on the protein fraction levels in alpacas; therefore, each piece of research adds knowledge to this subject. However, further research in this area should be conducted.

## Data Availability

Data will be made available on request to the corresponding author.
